# A lightweight hierarchical convolution network for brain tumor segmentation

**DOI:** 10.1186/s12859-022-05039-5

**Published:** 2022-12-13

**Authors:** Yuhu Wang, Yuzhen Cao, Jinqiu Li, Hongtao Wu, Shuo Wang, Xinming Dong, Hui Yu

**Affiliations:** 1 Department of Biomedical Engineering, Tianjin Key Laboratory of Biomedical Detecting Techniques and Instruments, Tianjin University, Tianjin, China; 2 Tianjin International Engineering Institute, Tianjin University, Tianjin, China; 3 Academy of Medical Engineering and Translational Medicine, Tianjin University, Tianjin, China; 4 Tianjin Rehabilitation Convalescent Center, Tianjin, China

**Keywords:** Brain tumor segmentation, Lightweight network, Deep learning, Convolutional neural network

## Abstract

**Background:**

Brain tumor segmentation plays a significant role in clinical treatment and surgical planning. Recently, several deep convolutional networks have been proposed for brain tumor segmentation and have achieved impressive performance. However, most state-of-the-art models use 3D convolution networks, which require high computational costs. This makes it difficult to apply these models to medical equipment in the future. Additionally, due to the large diversity of the brain tumor and uncertain boundaries between sub-regions, some models cannot well-segment multiple tumors in the brain at the same time.

**Results:**

In this paper, we proposed a lightweight hierarchical convolution network, called LHC-Net. Our network uses a multi-scale strategy which the common 3D convolution is replaced by the hierarchical convolution with residual-like connections. It improves the ability of multi-scale feature extraction and greatly reduces parameters and computation resources. On the BraTS2020 dataset, LHC-Net achieves the Dice scores of 76.38%, 90.01% and 83.32% for ET, WT and TC, respectively, which is better than that of 3D U-Net with 73.50%, 89.42% and 81.92%. Especially on the multi-tumor set, our model shows significant performance improvement. In addition, LHC-Net has 1.65M parameters and 35.58G FLOPs, which is two times fewer parameters and three times less computation compared with 3D U-Net.

**Conclusion:**

Our proposed method achieves automatic segmentation of tumor sub-regions from four-modal brain MRI images. LHC-Net achieves competitive segmentation performance with fewer parameters and less computation than the state-of-the-art models. It means that our model can be applied under limited medical computing resources. By using the multi-scale strategy on channels, LHC-Net can well-segment multiple tumors in the patient’s brain. It has great potential for application to other multi-scale segmentation tasks.

## Background

The brain tumor is one of the diseases that seriously threaten human life. According to statistics from the World Health Organization, the brain tumor is the second greatest cause of death from human diseases. The magnetic resonance image (MRI) has been extensively used in brain tumor detection and diagnosis [1]. MRI images have multiple modalities such as T1-weighted (T1), T2-weighted (T2), contrast-enhanced T1-weight (T1ce) and T2 Fluid Attenuated Inversion Recovery (Flair). These modalities show different contrasts for different brain tissues [2]. Accurate clinical brain tumor labels require tumor sub-regions including edema, enhancing tumor, necrosis and non-enhancing tumor. It is an important step for the treatment of patients [3]. However, manual annotation on each MRI image by doctors is inefficient, time-consuming and non-objective. Therefore, the automatic and accurate segmentation method has become the key instrument in clinical diagnosis and treatment.

In recent years, deep learning technology has been extensively studied and practiced [4, 5]. Many researchers have proposed some effective methods for medical images segmentation based on deep learning [6–8]. For brain tumor segmentation, semantic segmentation networks consisting of encoder and decoder are widely adopted. These networks are divided into 2D and 3D convolutional neural networks (CNNs).

The 2D network operates over 2D convolutions to perform 3D calculations on feature maps. Havaei et al. used a cascade network and solved the imbalance of brain tumor labels through the 2-phase training procedure [9]. Banerjee et al. combined the ConvNets with shortcut connections to improve the performance in locating and recovering object details [10]. Jungo et al. improved pooling flow and introduced residual flow in U-Net so that the network can combine global and local feature information [11]. These networks occupy fewer storage and computing resources. However, they ignore the continuity between slices, which is limited in extracting 3D spatial information. While the three-dimensional feature helps to improve the accuracy of brain tumor segmentation. Therefore, some researchers extracted features from different directions of a 3D volume [12, 13]. For example, Li et al. proposed a 2D network with three branches processing images along three directions: sagittal, coronal and axial views [14].

The 3D network operates over 3D convolutions to perform 4D calculations on feature maps. The spatial context information can be better combined by 3D input to improve the accuracy of segmentation. Isensee et al. achieved a competitive result by using nnU-Net trained on various types of medical images [15]. Myronenko et al. introduced a variational auto-encoder branch regularizing the encoder to reconstruct the MRI images for segmentation [16]. Xu et al. proposed DCAN with multiple branches, and each branch is responsible for a single target segmentation through a shared feature extractor [17]. Guan et al. proposed a combined segmentation network based on VNet, in which the Squeeze and Excite (SE) module is added to each encoder and the Attention Guide Filter (AG) module is added to each decoder [18]. Huang et al. proposed a deep multi-task learning framework which added a distance transform decoder based on the V-Net to improve the segmentation contour and reduce the generation of rough boundaries [19]. Zhang et al. reduced the difficulty of feature extraction by using multiple encoders, and they introduced a new loss function to solve the voxel imbalance problem [20]. Although the 3D network has higher segmentation accuracy than the 2D network, the 3D network is more complex than the 2D network. The parameters and the amount of calculation are greatly increased due to the additional dimension of expensive 3D convolutions. This makes it difficult to apply these models to medical equipment in the future. In addition, the 3D networks require a large scale of training data to prevent overfitting, while the amount of tumor image data with annotation is small due to the privacy of patients and the high cost of tumor image annotation [21]. Therefore, research for lightweight 3D networks is necessary for clinical applications to achieve high segmentation accuracy under limited medical computing resources.

Researchers have proposed many methods to reduce the model size. Chollet et al. designed the depthwise separable convolution operation [22]. Xie et al. [23], Szegedy et al. [24] and Gao et al. [25] introduced different grouped convolutions. Larsson et al. proposed the FractalNet with interacting subpaths of different lengths which achieved the effect of in-depth supervision [26]. These theories are applied to brain tumor segmentation networks. Nuechterlein et al. proposed the 3D ESP-Net with pyramidal refinement [27]. Chen et al. designed the S3D convolution module with three parallel branches to reduce the model parameters [28].

**Fig. 1:**
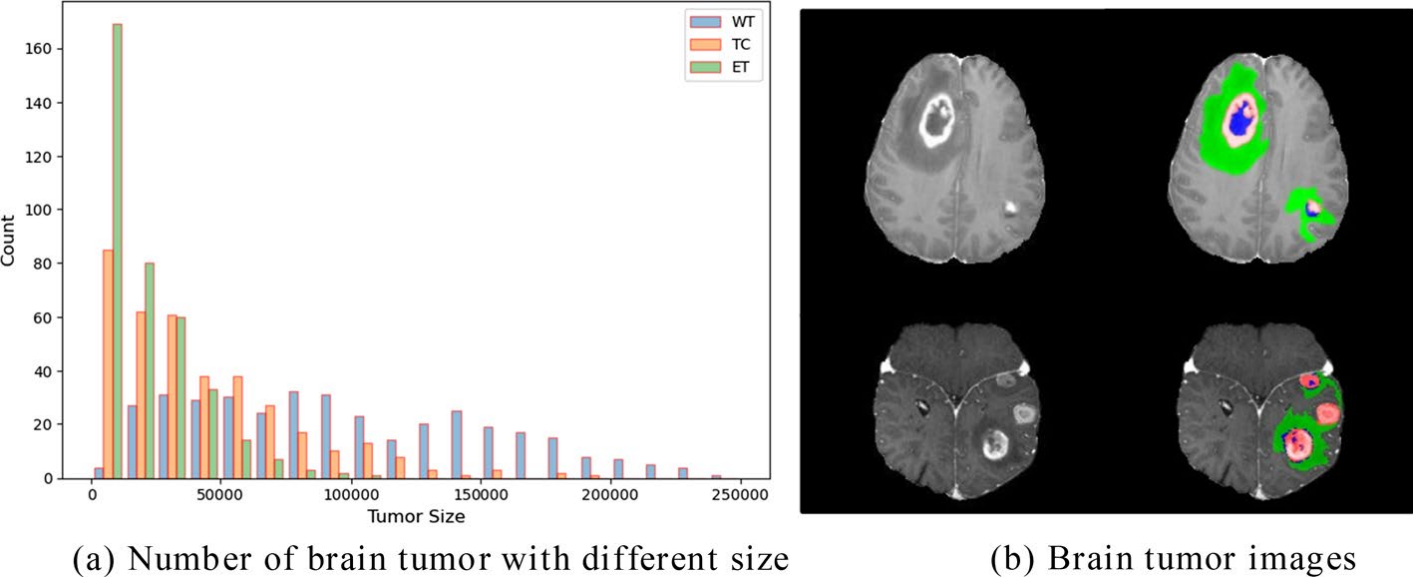
Distribution of brain tumor

The above methods have good results on the BraTS2020 datasets. However, these networks still need some improvement in their number of parameters and the amount of computation. We adopted the classic lightweight strategy of replacing standard convolutions with grouped convolutions. In addition, we counted the size of the whole tumor (WT), the tumor core (TC) and the enhancing tumor (ET), shown in Fig. 1a. We can see that the tumor size varies widely among patients. There are multiple tumors in the patient’s brain, as shown in Fig. 1b. Multi-scale feature representations of CNNs are of great importance for semantic segmentation [29]. The features of brain tumors of different sizes cannot be obtained simultaneously by using a single-scale CNN. Because if a single-scale CNN obtains a larger receptive field, it helps to extract the features of the whole tumor, while it will lose the information of the small-sized enhancing tumor and complex tumor borders. Likewise, if the receptive field is small, it is beneficial to extract the features of the tumor borders, while it is not conducive to the segmentation of the tumor as a whole. Therefore, we adopted a multi-scale strategy to extract brain tumor features. However, the classic multi-scale branching structure of Inception requires manual design and has large computation [29]. We decided to use Res2Net with hierarchical convolutions and residual-like connections [25]. The Res2Net adopts the group convolution strategy, which the different groups can get different receptive fields. It is beneficial to segment tumors of different sizes. Compared with other methods, our method achieves competitive segmentation performance with fewer parameters and less amount of computation.

The main contributions of this paper are as follows:

1. We designed a 3D RHC module, which can prevent the network from focusing too much on high-level features resulting in low segmentation accuracy.
2. We combined 3D U-Net, 3D Res2Net and 3D RHC to propose a lightweight hierarchical convolution network (LHC-Net) which can well-segment multiple tumors in the patient’s brain.
3. Our model is evaluated on the BraTS2020 and BraTS2018 public datasets, which shows that the model achieves the competitive segmentation results and has fewer parameters and less amount of computation than the state-of-the-art models.

## Methods

**Fig. 2:**
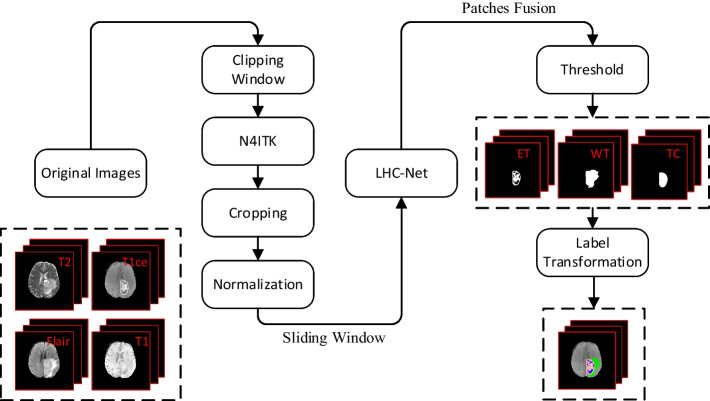
Overview of our proposed methods

An overview of our proposed method is shown in Fig. 2. Firstly, the clipping window and N4ITK algorithms are used to process original images [30]. For the processed images, most of the non-brain parts are cropped, and each modality is normalized using the zero-mean and unit-variance. Then, we designed a lightweight network based on the 3D U-Net. Our network uses multi-scale strategy to improve tumor segmentation performance and reduce parameters and computation. Finally, we adopted the sliding window and patches fusion to obtain a complete predicted probability map. The probability map is converted to the expected tumor sub-region label by threshold and label transformation.

### Preprocessing

**Fig. 3:**
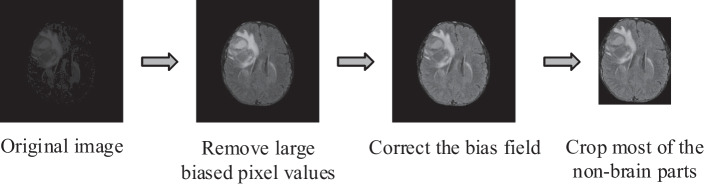
The preprocessing process for MRI images

The preprocessing process for MRI images is shown in Fig. 3. MRI images often show intensity non-uniformities due to variations in the magnetic field. As a result, there are some highly biased pixel values in the MRI images, and parts of the image may appear brighter or darker when visualized, simply because of variations in the magnetic field. The map of these variations is usually called the bias field. The bias field can cause problems for the segmentation performance of the model, as the variations in signal intensity are not due to any anatomical differences. Therefore, we clipped all brain voxel intensities with a window of [0.5–99.5%]. Specifically, the maximum and minimum values are computed by using the 99.5% and 0.5% percentiles based on the histogram, and these values are used to remove the large biased pixel values. Then we adopted N4ITK to correct the bias field [30].

The brain tumor semantic segmentation is a classification task for each pixel. In every sample, the brain volume is 10–20% of the entire sample volume and the whole tumor volume is less than 5%. To reduce the impact of class imbalance and the training scale, we processed each MRI sequence independently, in which most of the non-brain parts are cropped to obtain a small complete brain patch. Finally, the cropped brain patch is normalized by zero-mean and unit-variance normalization on each modality.

### 3D Res2Net module

**Fig. 4:**
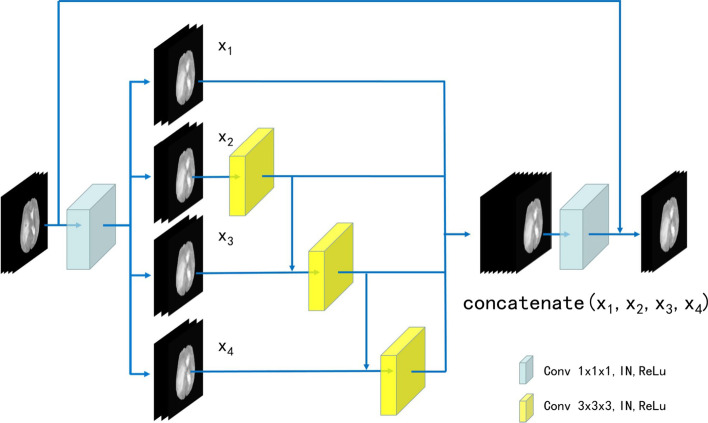
The view of 3D Res2Net module

Res2Net is a layer-wise CNN module that captures rich multi-scale features [25]. To obtain rich spatial information, we proposed 3D Res2Net module by adding a dimension to Res2Net module, as shown in Fig. 4. The common N-channel convolution filter within the residual block is divided into $s$ W-channel filters ($N = s \times W$), and the hierarchical residual-like connections are built within the module. The specific workflow of the 3D Res2Net module is that the input feature channels are increased through a 1 × 1 × 1 convolution filter. Then, the feature maps are equally divided into $s$ groups along the channel dimension and sent to 3 × 3 × 3 convolution filters. A group of input feature maps is processed through a filter. Another group of feature maps is concatenated with the output feature maps of the previous filter to be sent to the next filter. This process is repeated many times until all groups of input feature maps have been processed. Finally, the outputs of all filters are concatenated and sent to the second 1 × 1 × 1 convolution to fuse the feature maps. The operation equation is:

1 $$y_{i} = \left\{ {\begin{array}{*{20}l} {x_{i} ,} \hfill & {\quad i = 1} \hfill \\ {K_{i} (x_{i} ),} \hfill & {\quad i = 2} \hfill \\ {K_{i} (x_{i} + y_{i - 1} ),} \hfill & {\quad 2 < i \le s} \hfill \\ \end{array} } \right.,$$

where $s$ is the number of filters, $x_{i}$ is the i-th group of feature maps, $K_{i}$ is the convolution calculation and $y_{i}$ is the output of the filter. In the 3D Res2Net module, the feature maps are increased and decreased through two 1 × 1 × 1 convolutions, which improves the expression ability of feature information. The feature maps are divided into multiple groups and are processed by the hierarchical convolution, which reduce the amount of calculation. The hierarchical residual connection between the filters is beneficial to the network to extract multi-scale features.

### 3D RHC module

**Fig. 5:**
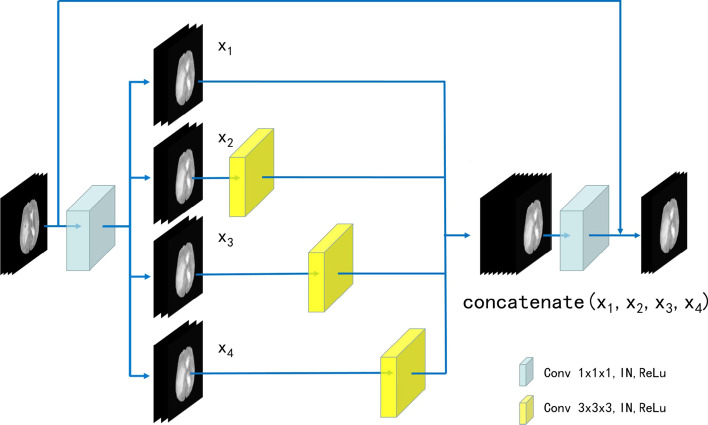
The view of the 3D RHC module

The study has shown that the first few layers of the network are more sensitive to noise [31]. The 3D Res2Net module extracts more complex features than common 3D convolutions under the same number of hidden units, which may lead to a decline in the ability to extract shallow features in the first few layers due to noise. Therefore, we designed the 3D residual hierarchical convolution (RHC) module, as shown in Fig. 5. The RHC module retains the hierarchical convolution and residual connection between input and output, and the hierarchical residual-like connections are removed in 3D Res2Net module. This change prevents the network from focusing too much on high-level features.

### LHC-Net

**Fig. 6:**
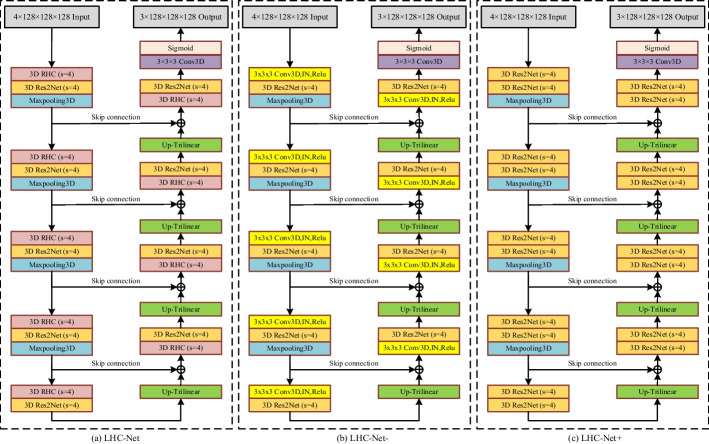
The view of our LHC-Net and its variants

Our proposed LHC-Net is shown in Fig. 6a. We adopted end-to-end multi-label learning to achieve pixel-level brain tumor segmentation. Images from multiple modalities are concatenated as the four-channel input to the network. The three-channel output is the predictions of WT, TC and ET, respectively. Because the tumor volume is small, especially the necrosis and non-enhancing tumor. Instead of directly predicting the three tumor sub-regions, our network predicts WT, TC and ET, and then these labels are translated into sub-regions by post-processing.

The backbone architecture of our network is the 3D U-Net. It consists of the encoder and the decoder. In the encoding path, the encoder uses maxpooling as the down-sampling layer. On the one hand, the down-sampling layers reduce the size of input data to one-half of the original size which expands receptive field of convolution to extract rich semantic features, such as high-level features. On the other hand, the down-sampling layers double the number of feature channels to express more semantic feature information. In the decoding path, the decoder uses trilinear interpolation as the up-sampling layer which restores the size of feature map to the same size as the expected labels. After up-sampling layers, the feature map is concatenated with the feature map from the encoding path through the skip connection. It combines the low-level and high-level semantic feature information to improve segmentation performance.

In the 3D U-Net, it is difficult to extract tumor features of different sizes at the same time by using common single-scale convolution. Therefore, we replaced the convolution with 3D Res2Net. The 3D Res2Net uses the multi-scale strategy on the channels to improve the multi-scale representation ability at a granular level. It is beneficial for the network to segment tumors of different sizes. On the other hand, for convolutional networks, the first few convolution layers usually extract low-level features. We usually increase the depth of the network to obtain high-level features required for pixel-label segmentation tasks. The down-sampling is repeated several times due to the limitation of storage capacity so that the resolution of the image is reduced. However, it causes that the last few convolution layers, which extract high-level features, are performed on low-resolution feature maps. By using the hierarchical convolutions and residual-like connections in 3D Res2Net, the feature maps are equally divided into several groups, and most of these feature map groups are processed more than once through the hierarchical convolution filter. Therefore, the first few layers can extract features at a higher level from high-resolution images. To prevent the network from focusing too much on high-level features, we combined 3D RHC and 3D Res2Net.

For ablation experiments, we also proposed two variants of LHC-Net: LHC-Net- and LHC-Net+, as shown in Fig. 6b and c, respectively. In the LHC-Net+, all convolutions in the 3D U-Net are replaced with 3D Res2Net which the network can extract more higher-level features at a fine-grained. In the LHC-Net-, half of the convolutions in each layer are replaced by 3D Res2Net.

### Complexity analysis

In this section, the complexity of LHC-Net is theoretically compared with 3D U-Net. The complexity of the model mainly depends on the number of parameters and floating point operations (FLOPs) [23, 24]. In the 3D U-Net, each convolution layer has two convolutions, the convolution kernel size is *k* × *k* × *k*, the number of input channels is $C_{in}$, output feature map size is $H_{out} \times W_{out} \times D_{out} \times C_{out}$. Without considering the bias, the number of parameters for each layer is:

2 $$Params_{U - Net} = 2 \cdot C_{in} \cdot k \cdot k \cdot k \cdot C_{out} .$$

In the LHC-Net, two 3D convolutions are replaced by a 3D RHC and a 3D Res2Net both with $s$ filters. Assuming that the number of feature map channels does not change through two 1 × 1 × 1 convolutions, the number of parameters for each layer is:

3 $$Params_{LHC - Net} = 2 \times \left[ {\frac{{C_{in} }}{s} \cdot k \cdot k \cdot k \cdot \frac{{C_{out} }}{s} \cdot (s - 1) + 2 \times 1 \times 1 \times 1 \cdot C_{in} \cdot C_{out} } \right].$$

If the kernel size is 3 × 3 × 3, the number of input and output channels are both 64, the number of filters within 3D RHC and 3D Res2Net are both 4. In the 3D U-Net, the number of parameters is 221184 in the convolution layer. While the number of parameters is 57856 in the LHC-Net. The input tensor of a 3D convolution is $C_{in} \times H_{in} \times W_{in} \times D_{in}$ and the output tensor is $C_{out} \times H_{out} \times W_{out} \times D_{out}$. Without considering the bias, the FLOPs of a 3D convolution is:

4 $$FLOPs = (C_{in} \cdot 2 \cdot k \cdot k \cdot k - 1) \cdot H_{out} \cdot D_{out} \cdot W_{out} \cdot C_{out} .$$

If the input image size is 4 × 128 × 128 × 128, the 3D U-Net has 148.17G FLOPs while the LHC-Net only has 35.58G FLOPs.

### Inference and post-processing

Due to the limited memory, we used a 128 × 128 × 128 sliding window with a 32 × 32 × 27 sliding step during the inference. Zero padding is used if the input cannot cover the sliding window. It means that the multimodal image is cropped into many patches as input to the network. Because there are many overlapping parts among output prediction patches, in order to obtain a complete three-channel brain tumor segmentation probability map, we used the patches fusion which these overlapping parts are averaged. Then, we used a fixed threshold, it is set as 0.5, to binarize the probability map so that we obtained the label of WT, TC and ET. Finally, according to these labels, we used the label transformation to obtain tumor sub-regions. It follows the rules: (a) the edema area is regarded as WT area minus TC area, (b) the necrosis and non-enhancing tumor is regarded as TC area minus ET area, and (c) the enhancing tumor is regarded as ET.

### Experiment settings

We randomly cropped patches of 128 × 128 × 128 from preprocessed images as input during the training phase. To improve the generalization ability, these patches are processed by online data augmentation including variance shift (− 0.1 to 0.1) with a probability of 0.2, intensity shift (0.9–1.1) with a probability of 0.2 and mirroring along three axes with a probability of 0.5. The training iteration has 450 epochs and the first five epochs are linear warmup. We used the Adaptive Moment Estimation (Adam) optimizer to train the network [32]. The initial learning rate is 0.001 and gradually decreases by $\left( {1 - epoch_{i} /epoch_{N} } \right)$. The batch size is four and the beta is (0.9, 0.999). The network is implemented in PyTorch 1.8 and is trained on GeForce GTX 3090 GPU.

### Evaluation metrics and loss function

The Dice similarity coefficient and the 95% Hausdorff distance (HD95) are used to evaluate the segmentation results which are sensitive to internal padding and borders, respectively. The equation of Dice similarity coefficient is:

5 $$D{\text{ice}}_{t} = \frac{{2\left| {P_{t} \cap T_{t} } \right|}}{{\left| {P_{t} } \right| + \left| {T_{t} } \right|}},$$

where $P_{t}$ is the positive prediction area of target $t$, such as $P_{WT}$, $P_{ET}$ or $P_{TC}$, and $T_{t}$ is the corresponding ground truth. |·| is the volume. The equation of Hausdorff distance is:

6 $$HD_{t} = d_{H} \left( {P_{t} ,T_{t} } \right) = \max \left\{ {d_{{P_{t} T_{t} }} ,d_{{T_{t} P_{t} }} } \right\} = \max \left\{ {\mathop {\max }\limits_{{x \in P_{t} }} \mathop {\min }\limits_{{y \in T_{t} }} d\left( {x,y} \right),\mathop {\max }\limits_{{y \in T_{t} }} \mathop {\min }\limits_{{x \in P_{t} }} d\left( {x,y} \right)} \right\},$$

where $P_{t}$ is the positive prediction area of target $t$, and $T_{t}$ is the corresponding ground truth. $x$ is a point on the surface $P_{t}$ and $y$ is a point on the surface $T_{t}$. $d(x,y)$ is the distance between $x$ and $y$. Because HD is highly susceptible to small outliers [3], we used HD95, which is the 95% quantile of HD.

In the training process, for the input with few or no annotated voxels, the small prediction errors may result in large gradients [33]. If the prediction errors are caused by under-fitting, the network is trained by these large gradients towards better segmentation. However, if the prediction errors are caused by imperfect annotations, which often occur at the tumor boundary, the network is trained in the wrong direction. In addition, these large gradients also cause instability in the training process. Therefore, we calculated the loss based on the statistics of all samples in a batch. The loss consists of soft dice loss and binary cross entropy loss (BCE loss). The equation is:

7 $$Loss = a \cdot Soft\;dice\;loss + b \cdot BCE\;loss,\quad a + b = 1,$$

where $a$ and $b$ are hyper-parameters, here $a = 0.2$ and $b = 0.8$. The equation of soft dice loss is:

8 $$Sof\;dice\;loss = \frac{1}{3M}\sum\limits_{b = 1}^{M} {\sum\limits_{t = 1}^{3} {\left( {1 - \frac{{2\left| {P_{b,t} \cap T_{b,t} } \right| + 1}}{{\left| {P_{b,t} } \right| + \left| {T_{b,t} } \right| + 1}}} \right)} } ,$$

where $M$ is the number of samples in a batch, $P_{b,t}$ is the prediction result of target $t$ of the b-th sample in a batch and $T_{b,t}$ is the corresponding ground truth. The equation of BCE loss is:

9 $$BCE\;loss = - \frac{1}{N}\sum\limits_{i = 1}^{N} {y_{i} \cdot \ln x_{i} + (1 - y_{i} ) \cdot \ln (1 - x_{i} )} ,\quad y_{{_{i} }} = 0{\text{ or }}1,$$

where $N$ is the number of pixels in a batch, $x_{i}$ is the predicted probability value of the i-th pixel and $y_{i}$ is the corresponding true value.

## Results

### Datasets

The datasets we used are BraTS2020 and BraTS2018 datasets which are collected by using different clinical protocols and various scanning instruments from 19 institutions [34–36]. BraTS2020 consists of a training set (369 samples), including 293 samples from glioblastoma (GBM/HGG) and 76 samples from lower-grade glioma (LGG), and a validation set (125 samples). BraTS2018 consists of 285 training samples and 66 validation samples. The datasets contain four modalities: T1, T2, T1ce and Flair. Each sample has a volume of 240 × 240 × 155. The brain tumor label consists of non-enhancing tumor and necrosis (NET/NCR-label 1), edema (ED-label 2) and enhancing tumor (ET-label 4).

According to the brain tumor label, we made the label of WT, TC and ET. ET is enhancing tumor. WT consists of NET/NCR, ED and ET. TC consists of NET/NCR and ET. These three labels, WT, TC and ET, are used for loss function and network evaluation. In the BraTS2020 training dataset, 20% (75 samples) of 369 samples are used as the test set and the remainder (294 samples) are used as the training set. The model was compared with others on BraTS2020 and BraTS2018 datasets.

### Comparison of LHC-Net with different hyper-parameters

LHC-Net uses the multi-scale strategy on the channels. This not only reduces the model parameters and computation, but also helps to extract the multi-scale features at a granular level. The more $s$ is, the more groups there are. It is beneficial for LHC-Net to obtain multi-scale receptive fields, while it weakens the connection between different feature groups. In order to ensure that each feature map input to the filter has the same number of channels. The values of $s$ are set to [1, 2, 4, 8]. The results are presented in Table 1. The ET Dice scores of $s > 1$ are significantly higher than $s = 1$. It means that the multi-scale strategy on the channels improves the multi-scale representation ability at a granular level. The LHC-Net with using $s = 4$ achieves the best Dice scores of 76.35%, 89.96% and 83.35% for ET, WT and TC, respectively. Therefore, we used $s = 4$ in the following experiments.

**Table 1:** Comparison of LHC-Net with different $s$ on the BraTS2020 test set

Methods	Dice (%)		
	ET	WT	TC
LHC-Net ($s = 1$)	73.12	89.13	81.52
LHC-Net ($s = 2$)	75.04	89.75	82.12
LHC-Net ($s = 4$)	**76.35**	**89.96**	**83.35**
LHC-Net ($s = 8$)	76.16	88.53	82.42

Bold indicates the best result for each evaluation metric

### Ablation of LHC-Net

An ablation study of LHC-Net on the BraTS2020 test set is presented in Table 2. There is a residual connection between each input feature and output feature in 3D Res2Net. Therefore, we investigated whether the residual connections alone improve segmentation performance. Compared with Res U-Net, LHC-Net- and LHC-Net+ both achieve performance improvement for ET by using the 3D Res2Net module. It shows that the segmentation performance is improved by hierarchical convolutions and residual-like connections, not just residual connections, especially for smaller targets. LHC-Net+ , which all standard convolutions are replaced by 3D Res2Net, achieves the highest Dice for ET while it gets a performance degradation for WT. It shows that LHC-Net+ pays too much attention to high-dimensional information. In addition, since the features with noise from the encoder or irrelevant features are directly processed by the 3D Res2Net in the decoder, it affects the performance of decoding at a fine-grained level. The LHC-Net achieves the best Dice scores for WT and TC while keeping segmentation performance for ET. It shows that 3D RHC module alleviates the difference in features between the encoder and the decoder.

**Table 2:** Ablation study of LHC-Net on the BraTS2020 test set

Methods	Dice (%)		
	ET	WT	TC
3D U-Net	73.50	89.42	81.92
Res U-Net	73.87	89.53	82.23
LHC-Net- (Conv3D + 3D Res2Net)	75.76	89.75	82.87
LHC-Net+ (3D Res2Net + 3D Res2Net)	**76.43**	89.36	82.20
LHC-Net (3D RHC + 3D Res2Net)	76.41	**90.05**	**83.34**

Bold indicates the best result for each evaluation metric

### Comparison with 3D U-Net and classic improved 3D U-Net

We trained the 3D U-Net and classic improved 3D U-Net under the same settings to evaluate our LHC-Net on the BraTS2020 test set. The results are presented in Table 3. The LHC-Net achieves the highest Dice scores for ET and TC. In particular, our model achieves significant performance on segmentation for ET, it shows that LHC-Net has a strong ability to extract fine-grained features. In addition, our model obtains a performance of 90.01% for WT, which is only 0.19% lower than 3D U-Net++. Compared with other networks, LHC-Net has fewer parameters and FLOPs, which is beneficial for application to medical equipment in the future.

**Table 3:** Comparison with 3D U-Net and classic improved 3D U-Net on the BraTS2020 test set

Methods	Dice (%)			HD95 (mm)			Params (M)	FLOPs (G)
	ET	WT	TC	ET	WT	TC		
3D U-Net	73.50	89.42	81.92	35.68	6.85	11.54	5.89	148.17
Res 3D U-Net	73.87	89.53	82.23	33.41	**6.19**	10.23	6.70	187.86
3D U-Net++	73.94	89.35	82.57	32.65	7.30	9.58	6.84	508.46
Attention 3D U-Net	74.42	**90.25**	82.86	30.24	6.72	9.35	6.47	151.51
LHC-Net (Ours)	**76.38**	90.01	**83.32**	**30.09**	6.96	**6.30**	**1.65**	**35.58**

Bold indicates the best result for each evaluation metric

**Fig. 7:**
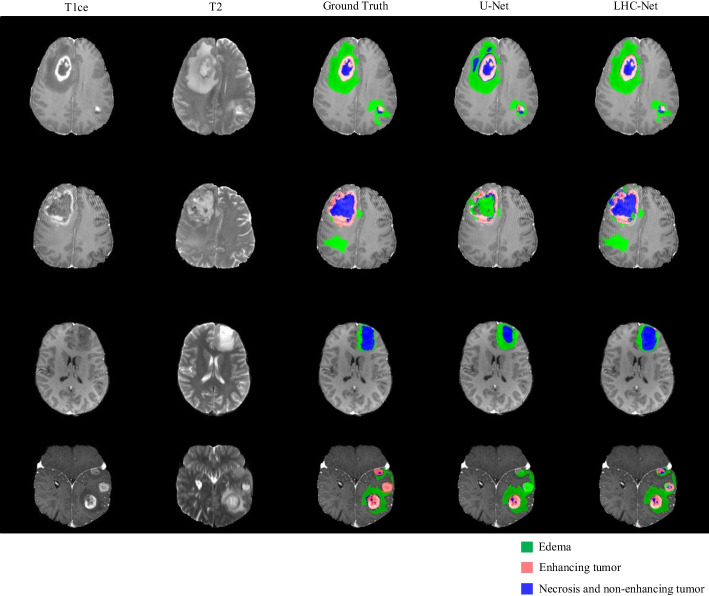
Predictions of LHC-Net and 3D U-Net on the BraTS2020 dataset

We visualized the segmentation results that are predicted by the 3D U-Net and LHC-Net on the BraTS2020 test set, shown in Fig. 7. Since the brain tumor varies widely among patients and there are multiple tumors in some patient’s brain, some methods cannot well-segment the tumors at the same time. LHC-Net can segment the tumors that are not segmented by 3D U-Net, and LHC-Net achieves better segmentation performance on ET and TC. In particular, LHC-Net can completely segment multiple tumors in a patient’s brain. It shows that LHC-Net has a better ability to extract multiple tumor features.

In addition, we divided the test set into a single tumor set (66 samples) and a multi-tumor set (9 samples) according to the number of tumors in the brain. In the multi-tumor set, each sample has at least two whole tumors or tumor cores in the brain. The comparison of segmentation results on these two sets is presented in Table 4. Due to false positive and false negative predictions for ET (see below for more detailed discussion), the Dice of ET is lower on the single tumor set than on the multi-tumor set. The Dice scores of TC and WT are lower on the multi-tumor set, which shows that it is difficult to segment multiple tumors at the same time. Compared with 3D U-Net, LHC-Net achieves Dice scores of 78.14%, 87.73% and 81.41% for ET, WT and TC in the multi-tumor set, which is the significant improvement. It shows that LHC-Net has good generalization performance.

**Table 4:** Comparison with 3D U-Net on the single tumor set and the multi-tumor set

Methods	Dice (%) the single tumor set (66)			Dice (%) The multi-tumor set (9)		
	ET	WT	TC	ET	WT	TC
LHC-Net	**76.14**	**90.32**	**83.58**	**78.14**	**87.73**	**81.41**
3D U-Net	73.30	90.31	82.41	75.00	82.92	78.33

Bold indicates the best result for each evaluation metric

### Comparison with the state-of-the-art methods

We compared the state-of-the-art methods for brain tumor segmentation on the BraTS2018 validation dataset, shown in Table 5. For LHC-Net, the Dice scores for ET, WT and TC are 76.82%, 90.21% and 83.79%, respectively. The Dice score for TC is highest, and the Dice scores for ET and WT are higher than most other methods significantly. It indicates that our methods can replace the manual segmentation of brain tumor. In addition, LHC-Net has 1.65M parameters and 35.58 FOLPs. It means that our methods are less demanding on hardware.

**Table 5:** Comparison with the state-of-the-art methods on the BraTS2018 validation dataset

Methods	Dice (%)			HD95 (mm)			Params (M)	FLOPs (G)
	ET	WT	TC	ET	WT	TC		
Kao et al. [37]	**78.75**	**90.47**	81.35	**3.81**	**4.32**	7.56	9.45	203.96
3D U-Net	75.26	88.69	80.55	4.51	11.34	8.07	5.89	148.17
S3D- UNet [28]	74.93	89.35	83.09	4.43	4.72	7.75	3.32	75.20
3D-ESPNet [27]	73.70	88.30	81.40	5.30	5.46	7.85	3.63	76.51
LHC-Net (Ours)	76.82	90.21	**83.79**	4.36	5.56	**6.79**	**1.65**	**35.58**

Bold indicates the best result for each evaluation metric

## Discussion

**Fig. 8:**
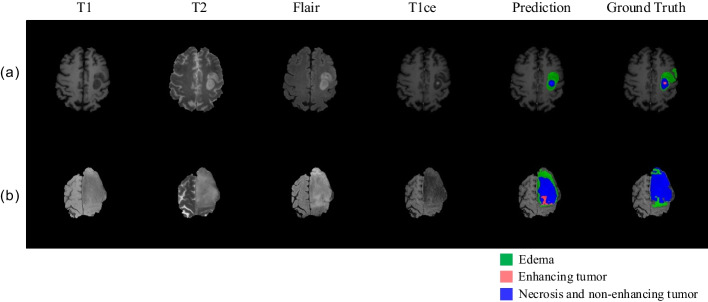
The two examples from outlier segmentation results with a Dice score of 0 for ET. **a** False positive prediction; **b** False negative prediction

In the segmentation results predicted by LHC-Net, there are seven cases with a Dice score of 0 for ET. Three of them are caused by false negative predictions, which ET is not found by the network, as shown in Fig. 8a. Four cases are caused by false positive predictions, which ET is incorrectly predicted, as shown in Fig. 8b. These seven cases show three characteristics: (a) the brain tumors are at the top of the brain, (b) ET is small or non-existent, (c) a greater proportion of NET/NCR in WT. We inferred that the cause of 0 Dice score for ET is sulcus. The sulcus shows a shadow on the image and is a relatively larger part at the top of the brain than other regions so that it is difficult to segment the small ET near the sulcus. These outlier segmentation results have a great influence on the scores of evaluation metrics. Therefore, in some studies [33, 38], in order to maximize the mean Dice score, a post-processing method is used, which enhancing tumor is entirely replaced by necrosis if the predicted volume is less than a certain threshold. It means that the correct prediction for the small enhancing tumor will be removed by the post-processing. We did not adopt this method because it ignores true positive predictions and increases false negative predictions which are more harmful to patients.

This study has some potential limitations. To improve the generalization ability, we used online data augmentation including variance shift (− 0.1 to 0.1) with a probability of 0.2, intensity shift (0.9–1.1) with a probability of 0.2 and mirroring along three axes with a probability of 0.5. However, the method may not be the best way to improve the generalization ability, and may not be ideal for all datasets. The data augmentation should be investigated further. In addition, for the proposed method, there should be no significant differences between training data and test data. The problem is common when applying deep learning methods to clinical medical practice. Moreover, the training data relies on a large number of 3D MRI images with pixel-level annotations. In the future, we will adopt a semi-supervised framework to reduce the manual annotation workload.

## Conclusion

Since the spatial information is very important for accurate brain tumor segmentation and most 3D networks are complex, we proposed a highlight brain tumor segmentation network based on 3D CNN. Because the size of the brain tumor is great difference and there are multiple tumors in some patient’s brain. To solve this problem, we used 3D Res2Net, which uses a multi-scale convolution strategy on the channels to obtain multi-scale receptive fields. It is beneficial for the network to well-segment tumors of different sizes. In addition, because the hierarchical residual-like connections in 3D Res2Net may cause the network to pay too much attention to high-dimensional information, we designed the 3D RHC. Finally, we combined 3D Res2Net, 3D RHC and 3D U-Net to propose LHC-Net. According to the experiment results on BraTS2020 and BraTS2018 datasets, our network has a better brain tumor segmentation performance than 3D U-Net, especially for multi-tumor. Compared to the state-of-the-art methods, LHC-Net has less parameters and less FLOPs while keeping competitive performance.

## Data Availability

The dataset used in our paper were available through https://ipp.cbica.upenn.edu/. This dataset requires permission from the Center for Biomedical Image Computing and Analysis at the University of Pennsylvania.

## Abbreviations

2D: Two-dimensional
3D: Three-dimensional
4D: Four-dimensional
BCE: Binary cross entropy
CNN: Convolutional neural network
MRI: Magnetic resonance imaging
FLOPs: Floating point operations
